# Infant Vocal Behavior During Contingent Vocal Imitation and Its Interruption as a Window Into the Emerging Sense of Agency

**DOI:** 10.1111/infa.70080

**Published:** 2026-03-09

**Authors:** Laura Diprossimo, Marlene Meyer, Caroline Lüdemann, Sabine Hunnius, Joscha Kärtner

**Affiliations:** ^1^ Department of Psychology University of Münster Münster Germany; ^2^ Joint Institute for Individualisation in a Changing Environment (JICE) University of Münster and University of Bielefeld Münster Germany; ^3^ Donders Institute for Brain Cognition and Behaviour Radboud University Nijmegen the Netherlands

**Keywords:** communication, imitation, responsiveness, sense of agency, social contingency, vocal development

## Abstract

Infants' emerging sense of agency is thought to be supported by caregivers' contingent responsiveness. However, it remains unclear which types of responses are most relevant to this process. Here, we examined the role of contingent vocal imitation, defined as the prompt repetition of an infant's vocalization by an interaction partner. To tease apart the contribution of contingent vocal imitation from other elements of social interactions, we developed a novel vocal contingency paradigm. First, we investigated whether 5‐ to 6‐month‐old infants could rapidly learn the contingency between their own vocalizing and a novel imitative response. Then, we examined whether infants tested this newly learned contingency when it was suddenly discontinued. Novel audio‐visual imitative responses were delivered and manipulated by an artificial agent. Infants' vocalizations were recorded while they experienced the novel contingency (connect phase) and its discontinuation (disconnect phase). Time‐course analyses indicated a significant linear increase in vocalization frequency over time in the connect phase, supporting the hypothesis that contingent vocal imitation enables rapid vocal contingency learning. Descriptively, data suggested a quadratic trend consistent with a vocal extinction burst during the disconnect phase. However, this trend did not reach statistical significance. Therefore, there was only partial support for the role of contingent vocal imitation in the emerging sense of agency (i.e., young infants quickly learned this contingency, but there was no evidence that they tested it upon discontinuation). Overall, our paradigm provided proof of concept that vocal contingency learning can be studied in the absence of a human interaction partner.

## 1 Introduction

Social contingency—the property of social interactions where the actions of one party occur shortly after those of another and are related to them (Social Contingency Consortium 2025)—is ubiquitous across cultures and species. In humans, social contingency is conducive to learning and development in a range of social‐cognitive and language domains (e.g., Bigelow et al. 2010; Bornstein and Tamis‐LeMonda, 1989; Goldstein et al. 2003, 2009; Goldstein and Schwade 2008; Ramírez et al. 2020). For instance, contingent speech models restructure the phonological patterns of 9.5‐month‐old infants' babbling (Goldstein and Schwade 2008); and interventions targeting parental contingent responsiveness from 6 months of age increase conversational turns and enhance infant vocabulary development at 18 months (Ramírez et al. 2020). Early in ontogeny, social contingency is thought to be the driver of a fundamental aspect of human development: the emergence of a *sense of agency* (Kärtner 2015). The sense of agency refers to the phenomenological experience of being in control of one's own actions, and, through them, events in the outside world (Haggard and Chambon 2012; Schlosser 2019). Indeed, it has been proposed that, during early caregiver‐infant interactions, caregivers' contingent responsiveness provides infants with unique opportunities to experience themselves as causal agents (e.g., Kärtner 2015).

Infants' vocalizations have received relatively little attention in the study of the emerging sense of agency under standardized paradigms (for an exception, see Bigelow and Power 2022), which has primarily focused on limb movements (Bednarski et al. 2022; Meyer and Hunnius 2021; Rovee and Rovee 1969; Zaadnoordijk et al. 2018, 2020). However, vocalizations are likely to play a crucial role in the emerging sense of agency because they are potent social signals. Across cultural contexts, infants' vocalizations reliably elicit timely responses from caregivers (Bornstein et al. 2015; Kärtner et al. 2008, 2010). Caregivers respond to about 30%–50% of infants' vocalizations during naturalistic interactions (e.g., Goldstein et al. 2003), and they are more responsive to vocalizations than non‐vocal signals, such as gaze and smile (e.g., Kärtner et al. 2008). Therefore, vocalizations are unique in eliciting high levels of contingent responsiveness from caregivers. Furthermore, early caregiver‐infant vocal exchanges are bidirectional and highly coordinated, taking the form of proto‐conversations or proto‐dialogs (Jaffe et al. 2001). For these reasons, vocalizations are likely to play a crucial role in the emerging sense of agency.

Infants have to acquire intentional vocal behavior before mastering intentional communication (E. Bates 1979; Bretherton and Bates 1979). Intentional communication is thought to develop gradually over the first year of life (Donnellan et al. 2020). We assume that a sense of agency in early vocal behavior, that is, feeling in control of vocalizations and their influence on caregivers' behavior, maps onto first‐order intentional communication and represents a foundational step along a continuum toward higher‐order intentionality (e.g., Townsend et al. 2017).

Contingent responsiveness is important for the emerging sense of agency, but which type of responses is most salient to infants? Caregivers' responses to infant vocalizations feature contingent vocal imitation of the sound produced by the infant (Masur and Olson 2008), or its prosodic contour (Gratier and Devouche 2011). Infants are sensitive and responsive to such contingent vocal imitation, which has a reinforcing effect on infant vocal behavior (Pelaez et al. 2011). We propose that this feature of early caregiver–infant interaction is particularly salient to the infant, given the high degree of correspondence between the two signals. Prior research in the action domain has also shown that infants process actions that mirror their own differently from those that do not. At the neurophysiological level, this is indicated by enhanced desynchronization of the EEG mu rhythm when infants observe an action that matches their own most recently executed action (Saby et al. 2012).

Against this background, the present study is the first to examine the role of contingent vocal imitation in the emerging sense of agency in infant vocal behavior. The remainder of this introduction provides a critical review of the relevant literature, followed by an outline of the study's research questions and hypotheses.

### 1.1 Sense of Agency in Infant Limb Movements

A wealth of prior research has investigated the emerging sense of agency using the well‐known mobile paradigm, which taps into infants' sensitivity to sensorimotor, non‐social, contingency, and was originally designed to investigate infant memory processes (Bednarski et al. 2022; Kelso 2016; Rovee and Rovee 1969; Zaadnoordijk et al. 2018, 2020). Under this paradigm, which standardly consists of a baseline, a connect, and a disconnect phase, movements of a limb trigger a visual effect (connect phase) that is subsequently discontinued (disconnect phase). Two specific changes in movement frequency across experimental phases and over time have been interpreted as indicators of the sense of agency, namely, a linear increase in movement frequency over time during the connect phase, and a further increase followed by a decrease in movement frequency during the disconnect phase, also known as *extinction burst*.

There is ongoing debate regarding the extent to which a sense of agency is present during the first months of life, and what constitutes a valid measure of this construct (Bednarski et al. 2022; Kelso 2016; Kollakowski et al. 2023; Zaadnoordijk et al. 2018). It has been noted that an increase in movement frequency over time in the connect phase could be simulated by an artificial agent lacking any causal action‐effect model (Zaadnoordijk et al. 2018). In light of this, the behavioral pattern observed during the disconnect phase seems to be more informative when it comes to assessing infants' sense of agency (Zaadnoordijk et al. 2018, 2020). Specifically, the extinction burst, an increase followed by a decrease in behavior frequency during the disconnected phase, was suggested to signal infants' attempts to test the lost contingency.

### 1.2 Sense of Agency in Infant Vocalizations

Somewhat surprisingly, infant vocalizations have received less attention compared to limb movements in the study of the emerging sense of agency under standardized paradigms. First indications come from the still‐face paradigm (Tronick et al. 1978). This paradigm typically consists of three face‐to‐face phases between the infant and an adult: A naturalistic interaction phase as a baseline, a still‐face phase in which the adult maintains a neutral expression and becomes unresponsive (resembling the disconnect phase described above), and a reunion phase of naturalistic interaction. Infants' non‐distress vocalizations and smiles while looking at an unresponsive partner, also known as social bids, have been interpreted as indicators of the sense of agency (Bigelow and Power 2022). Social bids during the still‐face phase have been shown to longitudinally increase from 1 to 2 months of age and to be predicted by maternal vocal contingency during naturalistic interactions (Bigelow and Power 2022). Prior research using the still‐face paradigm has also shown that 5‐ to 6‐month‐olds increase their rate of non‐cry vocalizations during the still‐face phase relative to the baseline (Delgado et al. 2002; Elmlinger et al. 2023; Franklin et al. 2014; Goldstein et al. 2009). Furthermore, the time course of non‐cry vocalization frequency within the still‐face phase resembles the rise‐and‐fall pattern of an extinction burst. For this reason, it has been dubbed *vocal extinction burst* 1 (Elmlinger et al. 2023; Goldstein et al. 2009). This behavioral pattern is thought to reflect infants' attempts to test and re‐establish contingent responsiveness, thereby indexing a sense of agency. Furthermore, naturalistic social contingency experience is concurrently related to the magnitude of the vocal extinction burst (Elmlinger et al. 2023), suggesting that caregiver responsiveness to infant vocalizations in everyday interactions supports the emergence of a sense of agency.

**FIGURE 1:**
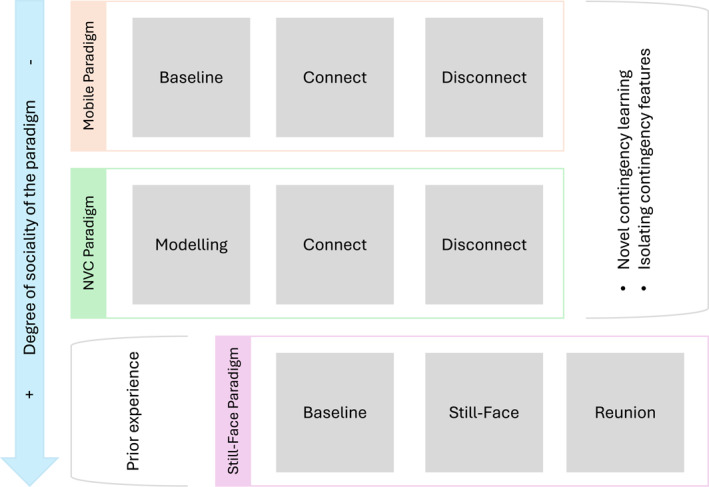
Schematic overview of paradigms tapping into the emerging sense of agency. From top to bottom: the mobile paradigm, the novel vocal contingency (NVC) paradigm, and the still‐face paradigm. The arrow indicates the degree of sociality involved in these paradigms, which is lowest in the mobile paradigm, highest in the still‐face paradigm, and in between for our newly developed NVC paradigm. We highlight the comparability of the disconnect phase with the still‐face phase in that it assesses infant reactions to the violation of expectations due to the sudden lack of responsiveness. We also note that the still‐face paradigm relies on the prior experience that the infant brings to the task, while the other paradigms tap into novel contingency learning.

As we compared the literature on the sense of agency using the mobile versus still‐face paradigm, several similarities and differences became apparent (see Figure 1 for a schematic overview). A crucial difference lies in the fact that the mobile paradigm taps into novel contingency learning, enabling us to assess moment‐to‐moment changes in infant behavior as a function of a new contingency's manipulation (i.e., presence vs. absence). In contrast, the still‐face paradigm relies on the prior experience that the infant brings to the task. Manipulating the presence of a *novel* vocal contingency is essential to examine contingency *learning* and to link specific contingency features to the moment‐to‐moment changes in vocal behavior. Only this approach can elucidate the elements and mechanisms that drive the sense of agency and vocal development in rich naturalistic social contexts over longer time scales.

To date, only a limited number of studies have systematically manipulated the presence of a novel vocal contingency to assess its effect on infant vocal behavior. One study has demonstrated that 6.5‐month‐old infants can learn the association between their vocalizing and a novel visual response (Keren‐Portnoy et al. 2021). However, this study did not examine infant vocal behavior upon the interruption of the newly learned contingency. Another study has investigated infant vocal behavior during both new contingency exposure and interruption (Venditti et al. 2024). In this study, 7‐month‐olds were shown to increase their rate of vocalizing when a newly learned audio‐visual contingency was interrupted. Yet, the contingent response provided in this study consisted of a pre‐recorded vowel sound, which had no relation to the sound produced by the infant. Thus, the role of contingent vocal *imitation* in this process has received little attention to date. This is surprising given the importance contingent vocal imitation might play for infants' emerging sense of agency. Against this background, we set out to probe, for the first time, the role of contingent vocal imitation on infant vocal behavior under a new, controlled paradigm that can illuminate moment‐to‐moment vocal contingency learning and infants' attempts to test such newly learned contingency when it is suddenly withdrawn.

### 1.3 The Present Study

The present study examined, first, whether 5‐ to 6‐month‐old infants can rapidly learn the contingency between their own vocalizing and a novel audio‐visual imitative response, and second, whether infants test this newly learned contingency when it is suddenly discontinued. Our research questions were as follows:

1. Do 5‐ to 6‐month‐old infants linearly increase the frequency of non‐cry vocalizations during a brief exposure to a novel audio‐visual imitative response delivered by an artificial agent?
2. Do 5‐ to 6‐month‐old infants exhibit a quadratic trend in the frequency of non‐cry vocalizations (i.e., vocal extinction burst) after the new audio‐visual imitative response is interrupted?

This age range was selected as it marks an important period in vocal development known as the expansion stage (Oller 2000). To address our research questions, we developed a Novel Vocal Contingency (NVC) paradigm and recorded infants' vocalizations while they were exposed to this new contingency (connect phase) and its discontinuation (disconnect phase). We hypothesized that, if infants quickly learn this association, their vocalization frequency should increase linearly during the connect phase (H1). If infants subsequently test this newly learned contingency upon experiencing its discontinuation, we should observe a quadratic trend in vocalization frequency consistent with a vocal extinction burst, during the disconnect phase (H2).

## 2 Methods

### 2.1 Transparency and Openness

Data and code necessary to reproduce the analysis presented here are available at the OSF project repository: https://doi.org/10.17605/OSF.IO/2BP3V. This study was not pre‐registered. The models were implemented in R version 4.1.3 (2022‐03‐10) with the function glmer of the R package lme4 (version 1.1–33) (D. Bates et al. 2015).

### 2.2 Participants

Five‐ to six‐month‐old infants were tested in a child‐friendly university lab, located in Münster, a medium‐sized city in Germany. The final sample consisted of *N* = 30 infants (*M*_age_ = 179.57 days, SD = 12.97, range = 157–204, 14 girls). Infants were born full term (> 37 weeks), healthy, and raised in German‐speaking environments. Concerning parity, 73.33% of infants were firstborn. The majority of the mothers reported living with their partner (93.33%). The average number of people per household was 3.30, with 0.39 siblings per participant on average. The average parental age was 33.62 years for mothers, and 36.33 years for their partners. Regarding parental education, a large proportion of mothers (80%) and their partners (67%) held a Bachelor's degree or higher. As a complementary index of socio‐economic status (SES), mothers also reported their perception of how comfortable the household lives on their current income on a 4‐point ordinal response scale (1 = living comfortably; 2 = getting by; 3 = having difficulties; 4 = having great difficulties). A sizable proportion of mothers reported living comfortably (46.67%) or getting by with their current household income (46.67%). Only a small proportion reported having difficulties associated with their income (6.67%). Taken together, parental education levels and perceived income difficulties indicate that our sample was skewed toward the higher end of the SES spectrum. The family migration background was also assessed. Mothers reported a migration background for their own parents (13.33%) or grandparents (13.33%), but did not migrate themselves (0%). They also reported a migration background for their partners (3.33%; *n* = 1 partner living in Germany for more than 10 years), their partners' parents (6.66%), or their partners' grandparents (6.66%). Information concerning race or ethnicity was not collected.

The sample size was established a priori based on prior studies of vocal learning in the same age group (Elmlinger et al. 2023; Keren‐Portnoy et al. 2021). In addition, we conducted a post‐hoc sensitivity analysis using a simulation‐based approach to determine the smallest effect size of interest (SESOI) that could be detected at a predetermined, conventional statistical power threshold (i.e., 80%), given our sample size and design (Kumle et al. 2021; Bolger and Laurenceau 2013). Analyses were conducted using the R package *simr* (version 1.0.8) (Green and MacLeod 2016). Results indicated that effects as small as 0.06 (coefficient expressed in log‐mean, given that the count outcome is modeled with a Poisson distribution) could be detected with 80% power.

Twenty‐five additional infants were tested but excluded from the present analysis because they showed signs of discomfort, such as prolonged fussing or crying, which resulted in the early interruption of the study paradigm (*n* = 24), or because they did not vocalize at all during the critical phase for contingency learning (*n* = 1). Participants' data were retained only if they contributed at least 1 min to the last phase of the paradigm. This resulted in a relatively high attrition rate (45%). However, this rate was comparable to studies using the still‐face paradigm (e.g., Elmlinger et al. 2023 observed a 44% attrition rate with 5‐month‐olds). We will return to this point in the Discussion. Written informed consent was obtained from the infants' caregivers prior to data collection. The study received ethical approval by the Ethics Committee of the Faculty of Psychology and Sports Science at the University of Münster, Germany (Reference Number: 2025‐13‐LD) and was conducted in full accordance with the ethical principles outlined in the Declaration of Helsinki. Infants were recruited in cooperation with the local city council, which provided the home addresses of newborns' guardians for the sole purpose of sending an invitation letter to the study via traditional mail. Participants were rewarded with a voucher for a local toy shop.

### 2.3 Set‐Up

**FIGURE 2:**
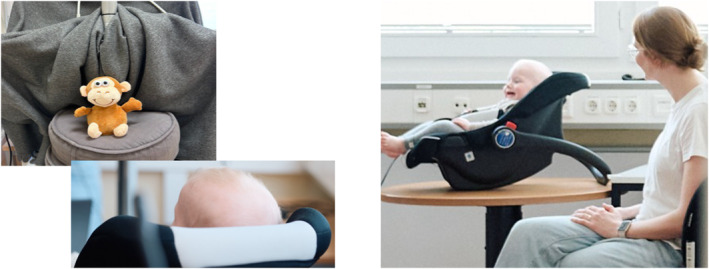
Overview of the set‐up. Photos of the contingent toy and participant in the observation laboratory.

The study took place in a child‐friendly observation laboratory. The session was recorded with two wall‐mounted video cameras (AXIS P5534 PTZ Dome Network Cameras), which were controlled remotely with the software MANGOLD Video Sync Pro and routed to a local computer via a video mixer (Behringer XENYX QX1202USB). Two microphones (t.bone GM 5212 Condenser gooseneck microphone) were placed at a distance of 60 cm from the infant and the contingent toy. The contingent toy (Figure 2) was a plush chatter‐monkey (brand: Kögler GmbH; model: 75611, size: 18 cm × 12 cm × 16 cm). When switched on, the toy provided contingent vocal imitation while also moving the head. The toy was placed on a pillow supported by a trolley located in front of the infant. The experimenter hid behind the trolley's curtains during the connect and disconnect phase.

### 2.4 Procedure

The procedure began with a short, naturalistic mother‐infant interaction (4 min), followed by the newly developed NVC paradigm (up to 7.5 min). The mother‐infant interaction served the purpose of getting the infant acquainted with the new environment and is not analyzed in the context of the current study. The data of interest for the current report are those collected during the NVC paradigm. Infants were seated in a car seat, which was secured on a table throughout the procedure. They were facing the toy during the NVC paradigm. The NVC paradigm consisted of a *modeling phase*, a *connect phase*, and a *disconnect phase*.

The modeling introduced the infant to the interactive toy, allowing to demonstrate its contingency early on without relying on the serendipitous advent of an infant vocalization. Preliminary piloting suggested that starting with a silent baseline would likely result in a higher attrition rate. During the modeling, a trained experimenter switched on the toy and held it close to the infant's sight to demonstrate its function. The experimenter followed a semi‐scripted interaction, producing short words and speech sounds (e.g., Hallo, ba–ba, wow) while alternating gaze between the infant and the toy, allowing time for the infant to join in. The modeling was adaptive and ended as soon as the infant produced the first vocalization, or after 2 min if no vocalization was produced. The modeling ended with the experimenter producing the word “gut” (good). The modeling was followed by the connect phase, where the experimenter hid behind the trolley's curtains and had the infant interact with the toy for 3 min without intervening. Then, the experimenter implemented the disconnect phase by switching the toy off while still hiding behind the trolley's curtains. The disconnect phase lasted for 2.5 min or until the infant showed prolonged signs of discomfort (such as fussing or crying).

During the connect and disconnect phase, the infant could not see the experimenter or the mother. This was done to prevent any confound that would arise from the adult's unintentional reactions to the infant's or toy's signals, and thereby to focus on the controlled manipulation we introduced. The order of phases was fixed as we were specifically interested in the effect of interrupting a newly learned contingency on infant vocal behavior. The experimenter used a stopwatch to keep the timing of the connect and disconnect phases constant. The length of the modeling was variable by design (and controlled for in our analyses). We expected that not all the infants would complete the last phase and decided a priori to include infants contributing at least 1 min to it. We assessed the fidelity of implementation, which was satisfactory. On average, the adaptive modeling lasted 66.93 s (SD = 40.34), the connect phase lasted 186.32 s (SD = 6.67), and the disconnect phase lasted 138.58 s (SD = 21.87).

### 2.5 Coding

We used an event coding approach and coded the onset and offset of each infant non‐cry vocalization (hereafter vocalization), using the coding software Mangold Interact (Version 18.5.5.1). Vocalizations were defined as all voiced sounds, excluding cries, effort sounds, and vegetative sounds such as sneezes, coughs, and hiccups (see Oller et al. 2013). A new onset was coded when there was a pause of more than 1 s between vocalizations. To assess inter‐rater reliability, one rater coded all the data, and a second rater coded 20% of the data. An intraclass correlation (ICC) on the aggregate level (i.e., frequency of vocalizations) was computed with the function ICC in R, specifying a two‐way model with absolute agreement (Wolak et al. 2012). The level of agreement for vocalization frequency was excellent during modeling (ICC = 1), connect (ICC = 0.99), and disconnect (ICC = 0.99), resulting in an excellent overall agreement (ICC = 0.99). We also coded the onset and offset of each toy response. During the connect phase, infant vocalizations elicited a toy response within 3 s from their offset 74% of the time. This is likely because low‐intensity vocalizations were not detected by the toy. Our modeling approach controlled for variation in the proportion of contingency experienced during the connect phase by each infant. We will return to this point in the Discussion.

### 2.6 Analytic Strategy

To assess whether infants rapidly learned the new vocal contingency and actively test it upon interruption, we modeled infants' vocal behavior during the connect and disconnect phase of our paradigm. First, we segmented the phases of our paradigm into 15‐s bins and computed the frequency of vocalizations in each bin for each subject. The 15‐s bin size was selected based on prior studies (Elmlinger et al. 2023). We excluded the last bin from the analysis if it lasted less than 13 s. This was to achieve an optimal trade‐off between comparability across bins and data inclusion. Following a similar logic, we retained bins contributed by at least 25 infants. This resulted in a total of 11 bins for the connect phase and a total of six bins for the disconnect phase (see Supporting Information S1: Figure S1 for a detailed description of the number of time bins contributed by each infant).

Following data preparation, we fit Generalized Linear Mixed‐Effects Models to predict vocalization frequency at the bin level. To evaluate the significance of the contribution of our test predictors, whilst avoiding multiple testing (Forstmeier and Schielzeth 2011), we compared our full model with a null model lacking our test predictors but being otherwise identical using the likelihood ratio test. The full model included the following test predictors: the fixed effects of phase of the paradigm (connect vs. disconnect; dummy coded with connect as reference), first‐ and second‐order polynomials of time bin, and the interaction of phase and bin polynomials. It controlled for gender (dummy coded with girls as the reference level) and the z‐transformed covariates of age (expressed in days), modeling duration (expressed in seconds), and proportion of toy contingency during the connect phase. The random effect structure included the random intercept of participant as well as the random slope of phase, to account for inter‐individual variation in overall response levels as well as in how individual infants respond to the phase condition. The null model lacked the test predictors but was otherwise identical.

We hypothesized that our full model would be a better fit for the data compared to the null model. Furthermore, we expected to observe a significant interaction between phase and bin polynomials. More precisely, we predicted a positive linear relationship between bin and vocalization frequency in the connect phase, indicating contingency learning (H1). We further expected a quadratic relation (i.e., inverted U‐shape, or rise and fall pattern) between bin number and vocalization frequency in the disconnect phase, that is, a behavioral pattern consistent with the vocal extinction burst, indexing an attempt to test the newly learned vocal contingency (H2). For our models, we report Incidence Rate Ratios (IRR), that is, exponentiated beta coefficients, as they allow for more intuitive interpretation of effect sizes.

## 3 Results

### 3.1 Descriptive Statistics

**FIGURE 3:**
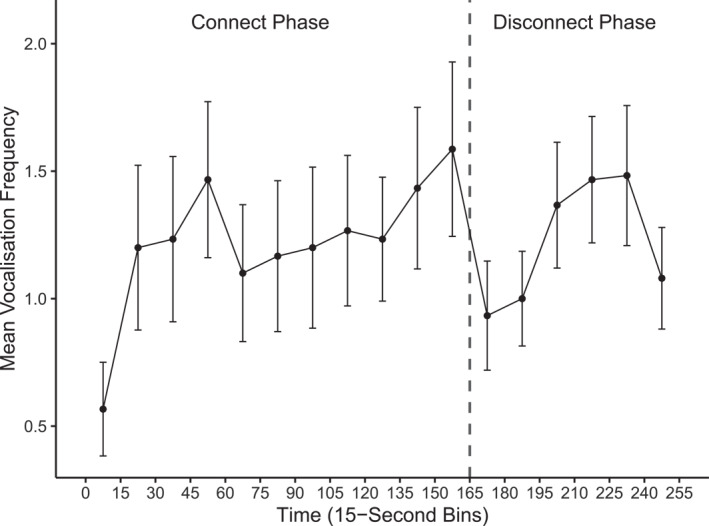
Mean vocalization frequency over time bins in the connect and disconnect phase. Mean vocalization frequency (*y* axis) over time bins (*x* axis) in the connect and disconnect phase (panels from left to right). Dots represent the mean values, and vertical error bars illustrate the standard error.

**FIGURE 4:**
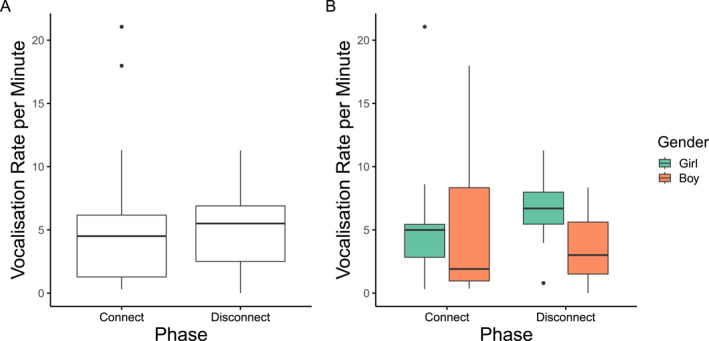
Boxplots illustrating the vocalization rate per minute by phase. (A) Boxplots illustrating the vocalization rate per minute (*y* axis) by phase (*x* axis). (B) Boxplots illustrating the vocalization rate per minute (*y* axis) by phase (*x* axis), by gender (color coded). The body of the box plot represents the interquartile range. The line that divides the box represents the *median* of the data. The lines extending from the box show the range of values representing the highest and lowest values, excluding potential outliers. Dots indicate potential outliers.

The mean vocalization frequency over time bins is depicted in Figure 3. We note that the mean vocalization frequency over 15‐s bins is comparable to that observed in the still‐face paradigm with 5‐month‐olds (Elmlinger et al. 2023). Individual infants' vocalization patterns are illustrated in Supporting Information S1: Figure S2. At the coarse‐grained level, the vocalization rate per minute by phase is illustrated in Figure 4. The mean vocalization rate per minute was 4.90 (SD = 5.10) in the connect phase, and 4.82 (SD = 3.17) in the disconnect phase.

### 3.2 Assessing Linear and Quadratic Trends in Vocalization Frequency

Model results are reported in Table 1. The likelihood ratio test indicated that our full model was a significantly better fit to the data compared to the null model (*χ* ^2^ = 16.318, df = 5, *p* = 0.006), supporting the explanatory value of phase and bin polynomials for vocalization frequency, above and beyond the contribution of demographic variables (i.e., age and gender) and methodological variables (i.e., duration of the adaptive modeling and proportion of toy contingency). Phase had a significant main effect on vocalization frequency (IRR = 1.53, CI = 1.04–2.26, *p* = 0.032), indicating that the vocalization frequency was higher in bins within the disconnect phase. The first‐order polynomial of bin was significantly and positively related to the vocalization frequency (IRR = 1.05, CI = 1.01–1.09, *p* = 0.011), indicating that, holding the other predictors constant at their reference level, the vocalization frequency linearly increased over time bins. However, model coefficients did not support the predicted interactions between phase and bin first‐ (IRR = 0.88, CI = 0.72–1.08, *p* = 0.212) and second‐order polynomial (IRR = 0.96, CI = 0.90–1.01, *p* = 0.130).

**TABLE 1:** Results of generalized linear mixed models predicting infants' vocalization frequency in 15‐second bins.

Predictors	Incidence rate ratios	SE	CI	*p*	Incidence rate ratios	SE	CI	*p*
(Intercept)	1.46	0.32	0.95–2.25	0.086	1.19	0.29	0.75–1.91	0.461
Gender (boy)	0.38	0.11	0.22–0.68	**0.001**	0.38	0.10	0.22–0.65	**< 0.001**
Age days *Z*	0.98	0.14	0.74–1.29	0.861	0.98	0.13	0.75–1.28	0.889
Dur modeling *Z*	0.73	0.12	0.53–1.00	0.050	0.75	0.12	0.55–1.01	0.059
Prop contingency *Z*	1.20	0.19	0.88–1.65	0.248	1.18	0.18	0.87–1.59	0.279
Bin phase (disconnect)					1.53	0.30	1.04–2.26	**0.032**
Bin number linear					1.05	0.02	1.01–1.09	**0.011**
Bin number quadratic					1.00	0.01	0.98–1.01	0.417
Bin phase (disconnect) × bin number linear					0.88	0.09	0.72–1.08	0.212
Bin phase (disconnect) × bin number quadratic					0.96	0.03	0.90–1.01	0.130
Random effects								
*σ* ^2^	0.79				0.79			
*τ*_00_	0.86_Child_ID_				0.92_Child_ID_			
*τ*_11_	0.59_Child_ID.Bin_Phasedisconnect_				0.56_Child_ID.Bin_Phasedisconnect_			
*ρ*_01_	−0.76_Child_ID_				−0.83_Child_ID_			
ICC	0.52				0.47			
*N*	30_Child_ID_				30_Child_ID_			
Observations	503				503			
Marginal *R* ^2^/conditional *R* ^2^	0.184/0.610				0.206/0.581			

*Note: σ* ^2^ = The variance of the residuals (errors) after accounting for both fixed and random effects; *τ*_00_ = The variance of the random intercepts, indicating the variability between subjects in their baseline levels; *τ*_11_ = The variance of the random slopes, reflecting the variability in the effect of a phase across subjects; *ρ*_01_ = The correlation between random effects components; ICC = The proportion of total variance attributable to the grouping structure in the data. *p*‐values lower than 0.05 are indicated in bold.

To inform our interpretation, we then ran phase‐specific models exploring linear and quadratic trends within each phase separately, following a similar full‐null model comparison strategy. The full models specified the bin first‐ and second‐order polynomials and the same control predictors as before. For the connect phase, we found that the full model was a significantly better fit to the data (*χ* ^2^ = 7.469, df = 2, *p* = 0.024). The first‐order polynomial of bin was positively and significantly related to the vocalization frequency (IRR = 1.05, CI = 1.01–1.09, *p* = 0.011), but this was not the case for the second‐order bin polynomial (IRR = 1.00, CI = 0.98–1.01, *p* = 0.425). These findings confirm the predicted linear increase in vocalization frequency over time bins in the connect phase. Model results are reported in Table 2.

**TABLE 2:** Results of generalized linear mixed models predicting infants' vocalization frequency in 15‐second bins in the connect phase.

Predictors	Incidence rate ratios	SE	CI	*p*	Incidence rate ratios	SE	CI	*p*
(Intercept)	0.92	0.23	0.56–1.51	0.742	0.91	0.24	0.55–1.52	0.729
Gender (boy)	0.66	0.24	0.33–1.34	0.250	0.66	0.24	0.33–1.34	0.250
Age days *Z*	0.88	0.17	0.61–1.27	0.489	0.88	0.17	0.61–1.27	0.491
Dur modeling *Z*	0.69	0.14	0.46–1.04	0.073	0.69	0.14	0.46–1.03	0.073
Prop contingency *Z*	1.34	0.27	0.91–1.99	0.143	1.34	0.27	0.91–1.99	0.142
Bin number linear					1.05	0.02	1.01–1.09	**0.011**
Bin number quadratic					1.00	0.01	0.98–1.01	0.425
Random effects								
*σ* ^2^	0.86				0.86			
*τ*_00_	0.71_Child_ID_				0.70_Child_ID_			
ICC	0.45				0.45			
*N*	30_Child_ID_				30_Child_ID_			
Observations	329				329			
Marginal *R* ^2^/conditional *R* ^2^	0.206/0.564				0.214/0.568			

*Note: σ* ^2^ = The variance of the residuals (errors) after accounting for both fixed and random effects; *τ*_00_ = The variance of the random intercepts, indicating the variability between subjects in their baseline levels; *ρ*_01_ = The correlation between random effects components; ICC = The proportion of total variance attributable to the grouping structure in the data. *p*‐values lower than 0.05 are indicated in bold.

For the disconnect phase, we found that the full model was a significantly better fit to the data compared to the null model (*χ* ^2^ = 6.409, df = 2, *p* = 0.040). However, the first‐ and second‐order polynomials of bin were not statistically significant. Model results are reported in Table 3. We note that an exploratory model where the linear term was removed supported the quadratic trend (IRR = 0.97, CI = 0.95–0.99, *p* = 0.017; see Supporting Information S1: Table S1). However, the results of such an exploratory model should be interpreted with caution.

**TABLE 3:** Results of generalized linear mixed models predicting infants' vocalization frequency in 15‐second bins in the disconnect phase.

Predictors	Incidence rate ratios	SE	CI	*p*	Incidence rate ratios	SE	CI	*p*
(Intercept)	1.66	0.28	1.19–2.32	**0.003**	1.91	0.35	1.33–2.72	**< 0.001**
Gender (boy)	0.36	0.10	0.22–0.61	**< 0.001**	0.36	0.10	0.21–0.61	**< 0.001**
Age days *Z*	0.99	0.13	0.77–1.28	0.966	0.99	0.13	0.77–1.28	0.968
Dur modeling *Z*	0.77	0.11	0.57–1.03	0.079	0.77	0.11	0.57–1.03	0.077
Prop contingency *Z*	1.17	0.18	0.87–1.58	0.298	1.17	0.18	0.87–1.59	0.294
Bin number linear					0.93	0.09	0.76–1.14	0.487
Bin number quadratic					0.95	0.03	0.90–1.01	0.100
Random effects								
*σ* ^2^	0.72				0.72			
*τ*_00_	0.26_Child_ID_				0.26_Child_ID_			
ICC	0.27				0.27			
*N*	30_Child_ID_				30_Child_ID_			
Observations	174				174			
Marginal *R* ^2^/conditional *R* ^2^	0.259/0.458				0.279/0.472			

*Note: σ* ^2^ = The variance of the residuals (errors) after accounting for both fixed and random effects; *τ*_00_ = The variance of the random intercepts, indicating the variability between subjects in their baseline levels; *ρ*_01_ = The correlation between random effects components; ICC = The proportion of total variance attributable to the grouping structure in the data. *p*‐values lower than 0.05 are indicated in bold.

Taken together, our analyses consistently detected a linear increase in vocalization frequency in the connect phase (H1) but did not support the predicted quadratic trend in the disconnect phase (H2). With respect to control predictors, we note that our analyses consistently detected gender differences, indicating that baby boys were less likely to vocalize than baby girls. Phase‐specific analyses and visual exploration suggest that such gender differences might be driven by the disconnect phase. The control predictors of age, modeling duration, and proportion of contingency were not significant across models.

Because some prior research has also modeled vocal behavior at a coarse‐grained level in terms of vocalization rate per minute in each phase (Elmlinger et al. 2023; Goldstein et al. 2009; Venditti et al. 2024), we also ran such analyses for comparability (see Supporting Information S1: Table S2). These analyses revealed a similar effect of control predictors, but no main effect of phase, suggesting that, at least in our study, aggregating vocal behavior at a too coarse‐grained level may obscure some meaningful patterns.

Visual exploration indicated that infants started off with a relatively low vocalization frequency in the first bin of the connect phase (see Figure 3 and Supporting Information S1: Figure S2). Therefore, we run an exploratory model to assess how excluding the first bin of the connect phase would affect our main results. The effect of phase remained significant and of identical size after excluding the first bin of the connect phase (Supporting Information S1: Table S5). However, in this exploratory model, the linear polynomial term was no longer significant, and the effect size was reduced. This indicates that including the relatively low vocalization frequency at the start of the connect phase is necessary to detect a linear increase within this phase.

## 4 Discussion

This study investigated whether infants aged 5–6 months can rapidly learn the contingency between their own vocalizing and a novel audio‐visual imitative response, and whether they test this newly learned contingency when it is suddenly discontinued. The results indicate a significant linear increase in vocalization frequency over time only when the contingency was available. This supports our hypothesis that contingent vocal imitation enables young infants' rapid vocal contingency learning. However, the predicted quadratic trend, which would suggest that infants test this newly learned contingency after its discontinuation, was only detected at a descriptive level and could not be confirmed statistically.

The linear increase in infant vocalization frequency during exposure to contingent vocal imitation indicates that infants were capable of rapid contingency learning. This is in line with our prediction and extends prior research in important ways. For instance, this result extends prior findings indicating that contingent maternal vocal imitation serves as a reinforcement for infant vocalizations (Pelaez et al. 2011). It also provides an important complement to observational research on caregiver‐infant interaction, which has long indicated a high degree of bidirectional vocal coordination between caregivers and young infants (Jaffe et al. 2001). Here, we demonstrated that, in the absence of and without any interference from another human being, 5‐ to 6‐month‐old infants respond to contingent vocal imitation by increasing the frequency of vocalizing within just 3 min. This finding suggests that young infants are highly sensitive to contingent vocal imitation and that this contingency feature may play an important role in the development of a sense of agency.

One of the novelties of our study is that the contingency was both imitative and controlled by a non‐human entity, which allowed us to extract the elements that might be underlying effects observed in richer social interactions. Compared to studies where a non‐imitative vocal contingency is manipulated by a non‐human entity, we found evidence of contingency learning with less exposure: 3 min in our study versus 20 min in Venditti et al. (2024). Furthermore, we were able to detect such rapid contingency learning in a relatively young sample: 5‐ to 6‐month‐olds in our study versus 7‐ to 8‐month‐olds in Venditti et al. (2024). We speculate that an imitative response is particularly salient and thus enables rapid learning in younger infants—an assumption that warrants further empirical testing. More broadly, these findings support the theoretical stance that the social environment and its affordances, such as contingent vocal imitation, play a crucial role in communicative development (Bruner 1983; Tomasello 2003; Vygotsky 1978).

One might wonder whether the linear increase detected in the connect phase could be explained by natural fluctuation in infant vocal behavior, and particularly whether starting the connect phase after the infant produced the first vocalization could have biased our results, because that would signal the onset of a vocal bout. While our design does not allow us to fully rule out this possibility, we think that this alternative interpretation is unlikely. First, prior research suggests that, during short, lab‐based dyadic interaction between caregivers and their 5‐month‐olds, vocal turn‐taking bouts have an average frequency of 0.76 per minute (SD = 0.55), and an average length of 4.17 turns (SD = 1.11) (Zhang et al. 2024). If we take this as a benchmark, it seems unlikely that the linear increase we detected in the 2‐min connect phase is an artifact due to the onset of a vocal bout, because that would typically be much shorter than 1 min. In addition, the width of our time bins was 15 s (Elmlinger et al. 2023; Goldstein et al. 2009). Therefore, even if the onset of the connect phase coincided with the beginning of a vocal bout, this would be unlikely to create a systematic linear increase across the entire connect phase. If anything, starting the phase at a moment of heightened vocal activity would bias the pattern against the detection of an increasing trend.

We did not find evidence of a vocal extinction burst indicating an active attempt to test the newly learned contingency when it was discontinued. The absence of a detectable vocal extinction burst in our data is compatible with several possible interpretations. One possibility is that an extinction burst did occur, but limitations in statistical power or the granularity of our temporal analysis precluded its detection. This raises the broader inferential issue of whether our findings reflect genuine evidence for the absence of an effect, or rather an absence of evidence due to measurement constraints. The observation of a rise‐and‐fall pattern at the descriptive level, along with the results of an exploratory model (see Supporting Information S1: Table S3), supports the latter interpretation. Additionally, given the variability in the temporal expression of extinction bursts across individuals (see Supporting Information S1: Figure S2), it is plausible that divergent timing of peak response rates among infants obscured group‐level effects.

Alternatively, the extinction burst may have been truly absent as a consequence of the structure or timing of the experimental paradigm. It is important to note that the vocal extinction burst observed in the context of the still‐face paradigm (Delgado et al. 2002; Elmlinger et al. 2023; Franklin et al. 2014; Goldstein et al. 2009) likely results from the violation of expectation about social contingency that infants have accumulated during social interactions over the previous months of life. This prevents us from assessing contingency learning in the moment due to the very nature of this paradigm, which does not involve any exposure to a novel contingency. When the presence of a novel vocal contingency is manipulated, 20 min of exposure to social responsiveness is sufficient to elicit a vocal extinction burst (Venditti et al. 2024). In light of these observations, future work should extend the duration of exposure to new contingencies to establish the minimal dosage and optimal learning schedule to develop a predictive model that will ultimately result in a vocal extinction burst. There may be practical constraints in extending the length of contingency exposure while maintaining a controlled setting, as we have done in the current study. In prior studies, adults were visible to support infant engagement (Keren‐Portnoy et al. 2021; Venditti et al. 2024). Future research should consider the trade‐off between feasibility and internal validity.

Another possibility pertains to the role of motivation and reward in the expression of the vocal extinction burst. Indeed, one might assume that infants would test whether a contingency is lost if they are motivated to do so, for instance, because they find this contingency rewarding. The artificial agent was selected under the assumption that it would be engaging for infants. Indeed, beyond its cuddling toy appearance, the agent featured contingent vocal imitation, coupled with self‐propelled motion. Contingency has been linked to the formation of social expectations about artificial agents in young infants (Venditti et al. 2024), and self‐propelled motion allows infants to discriminate between animate and inanimate entities (Markson and Spelke 2006). Despite this, it remains possible that infants in our study were not motivated enough to test this contingency when it was interrupted, purely because of insufficient motivational engagement with an artificial agent as compared to a human being.

Finally, we cannot rule out the possibility that 5‐ to 6‐month‐olds were just too young to display a vocal extinction burst. However, our selection of age group was grounded on prior empirical evidence indicating a vocal extinction burst at 5 months under the still‐face paradigm (Elmlinger et al. 2023; Goldstein et al. 2009), as well as 6‐month‐olds' ability to rapidly learn a new vocal contingency (Keren‐Portnoy et al. 2021).

Regarding our control predictors, our analyses also identified gender differences, with boys being significantly less likely to vocalize than girls. While this effect was not the focus of our research, and therefore should not be interpreted as confirmatory, it is important to note it and discuss it. The identified gender differences in infant vocalization frequency echo prior evidence documenting girls' advantage in early expressive language development between 6 and 36 months (Frank et al. 2021). However, it is worth noting that research on early vocal development in the first year of life has challenged this notion: Baby boys are found to produce more proto‐phones than baby girls (Oller et al. 2020). Given these contradictory findings, future research in vocal learning should, at the very least, control for gender to provide unbiased estimates of the effects of any manipulation. In our study, the gender differences seemed to be driven by the behavior rate in the disconnect phase. This underscores the importance of exploring the source of any gender differences in the development of a sense of agency, particularly in relation to the relative contributions of the infant and their caregiver, in line with a recent theoretical framework emphasizing the role of child and caregiver characteristics in the scaffolding process (Carranza‐Pinedo and Diprossimo 2025). Furthermore, male infants showed higher inter‐individual variability in the vocalization rate during the connect phase than female infants. This variability might suggest greater heterogeneity in their sensitivity to or motivation for engaging in contingent vocal exchanges. Such findings could also reflect the greater inter‐individual variability in temperament typically observed in boys (Else‐Quest et al. 2006). Further research is needed to systematically explore whether these early differences are stable and predict later social–communicative development in larger samples, to provide potential insights into the sources of the well‐documented gender differences in early vocabulary size (Frank et al. 2021).

Age‐related differences were not found, and they were not the focus of this study, which targeted a relatively narrow age range. Future studies, sampling a broader age range in a cross‐sectional or longitudinal fashion, are clearly needed to document the developmental trajectory of the sense of agency and vocal learning abilities using similar paradigms. Our methodological control predictors, namely the duration of the adaptive modeling and the proportion of contingent responses delivered by the toy, were not significant.

### 4.1 Limitations and Future Directions

This study comes with limitations and suggestions that should be considered in future research. First, there was a relatively high attrition rate (45%), which raises the possibility that our findings may not generalize across the temperament spectrum, for instance, to infants who are more prone to fussiness in novel or standardized settings. Second, the commercial toy we used to implement the contingency manipulation provided reasonable but imperfect fidelity of implementation. It responded contingently to 74% of infant sounds, likely because low‐intensity sounds were not detected. While this is close to contingency rates in caregiver‐child interactions, we acknowledge this is a potential limitation of our study. We addressed this limitation with two complementary approaches: We controlled for the proportion of contingent responses delivered by the toy to each infant in our main analyses and found that this predictor was not significant. Furthermore, as a robustness check, we reran our analyses excluding participants (*n* = 6) who experienced a low contingency proportion (< 25%), but this did not alter the pattern of the results (see Supporting Information S1: Table S4).

Another potential limitation is that our design cannot rule out the presence of order effects. However, a fixed order was justified by our research question targeting infants' attempts to test a newly learned contingency. Including a yoked control condition is a necessary next step to rule out alternative interpretations, such as non‐specific effects of vocal stimulation, regardless of whether it is contingent on infant vocal behavior. Furthermore, a direct contrast of contingent imitation with a contingent but non‐imitative control condition is necessary to draw firmer conclusions about the unique role of imitation. The insights generated by the current study can inform this critical research endeavor. Specifically, we have shown that it is possible to study vocal contingency learning in a highly controlled setting, where the contingency is delivered by a non‐human entity. This opens up important avenues for future research to examine the relative contribution of different contingency features to moment‐to‐moment changes in vocal behavior, without any confound that may arise from humans' unintentional reactions to infants' signals. This line of work is an important complement to naturalistic observation, as it can advance our understanding of the underlying mechanisms driving the development of the sense of agency and vocal learning in naturalistic settings over longer time scales. We note that the use of an artificial agent enhances the internal validity of our findings but comes at the cost of reduced external validity. A converging evidence approach and careful consideration of the trade‐off between experimental control and ecological validity are essential to advancing this line of inquiry.

A final, broader limitation concerns the measurement of pre‐verbal infants' phenomenological experiences. Measuring a sense of agency in infancy is a fascinating yet extremely challenging research endeavor. We devoted particular attention to the vocal extinction burst during the disconnect phase, because this behavioral pattern (contrary to the one observed in the connect phase) cannot be explained by a learning mechanism lacking a cause‐and‐effect representation (Zaadnoordijk et al. 2018). We acknowledge that in some cases, the extinction burst has been observed in non‐human animal research as a result of extinguishing the reward in the context of reinforcement learning paradigms (Ferster and Skinner 1957; Katz and Lattal 2020). Therefore, this index should be complemented by other measures to rule out lower‐level explanations that do not require any sense of agency.

### 4.2 Conclusions

Our findings support the prediction that contingent vocal imitation has the power to shape young infants' moment‐to‐moment vocal behavior: Within just 3 min, infants responded to a vocally contingent non‐human agent by linearly increasing their frequency of vocalizing. We conclude that young infants are highly sensitive to contingent vocal imitation, which bears important theoretical and practical implications: This feature of early interaction deserves further empirical attention and might be exploited for translational purposes. Contrary to our prediction, however, there was no conclusive evidence that infants tested the newly learned contingency once interrupted. For such null findings, there are multiple possible explanations that can only be teased apart by future studies. Future work might also usefully seek to extend the findings presented here by comparing different exposure schedules, contingency features, contexts, and age groups. This will allow for a more nuanced understanding of infants' developing sensitivity to the timing and structure of social contingency and its links with the sense of agency and vocal development.

## Author Contributions

**Laura Diprossimo:** conceptualization, investigation, funding acquisition, writing – original draft, methodology, validation, visualization, writing – review and editing, formal analysis, project administration, data curation, supervision. **Marlene Meyer:** conceptualization, methodology, writing – review and editing, supervision. **Caroline Lüdemann:** conceptualization, investigation. **Sabine Hunnius:** conceptualization, methodology, writing – review and editing, supervision. **Joscha Kärtner:** conceptualization, methodology, writing – review and editing, supervision, resources.

## Ethics Statement

This study has received ethical approval from the Ethics Committee of the Faculty of Psychology and Sports Science at the University of Münster, Germany (Reference Number: 2025‐13‐LD) and was conducted in full accordance with the ethical principles outlined in the Declaration of Helsinki.

## Conflicts of Interest

The authors declare no conflicts of interest.

## Supporting information


Supporting Information S1

## Data Availability

Data and code necessary to reproduce the analysis presented here are available at the OSF project repository: https://doi.org/10.17605/OSF.IO/2BP3V. This study was not pre‐registered.

## Endnotes

^1^
The term vocal extinction burst is used somewhat interchangeably in the literature to refer to the characteristic rise and fall pattern within the still‐face phase, as well as the increase in vocalization rate during the still‐face phase relative to baseline.
