# Supplementary material for: Infant Vocal Behavior During Contingent Vocal Imitation and Its Interruption as a Window Into the Emerging Sense of Agency
Source: Infancy. 2026 Mar 9;31(2):e70080. doi: 10.1111/infa.70080 (PMC12971622; doi:10.1111/infa.70080)
Supplement: Supplementary file 1 — Supporting Information S1 [file INFA-31-0-s001.pdf]

Supplementary Materials

Figure S1A illustrates the number of bins completed by each infant in each phase. To ensure comparability across bins, and also to prevent biases that may arise from modelling bins with a smaller sample size, we included bins with a sample size of at least 25 infants in our primary analyses. Figure S1B illustrates the bins included in our main analyses. As a robustness check, we re-run our analyses including all bins and found the same pattern of results (see Table S3)

Figure S1. Number of Bins Completed (A) and Included (B) Per Infant by Phase (Colour-Coded)

A

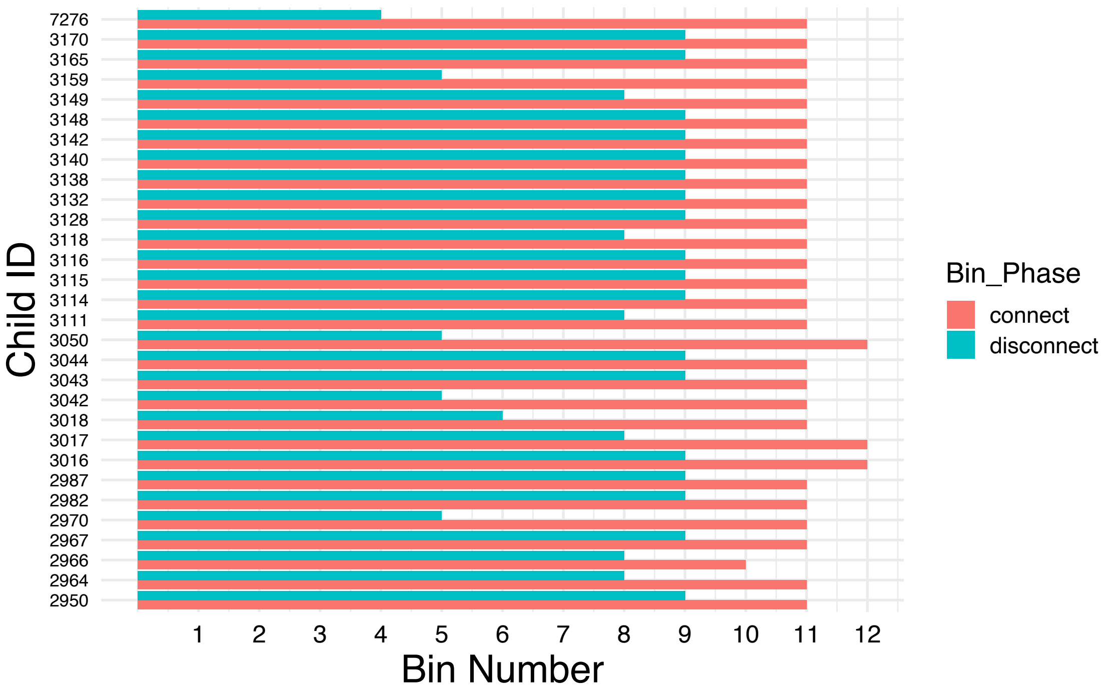

B

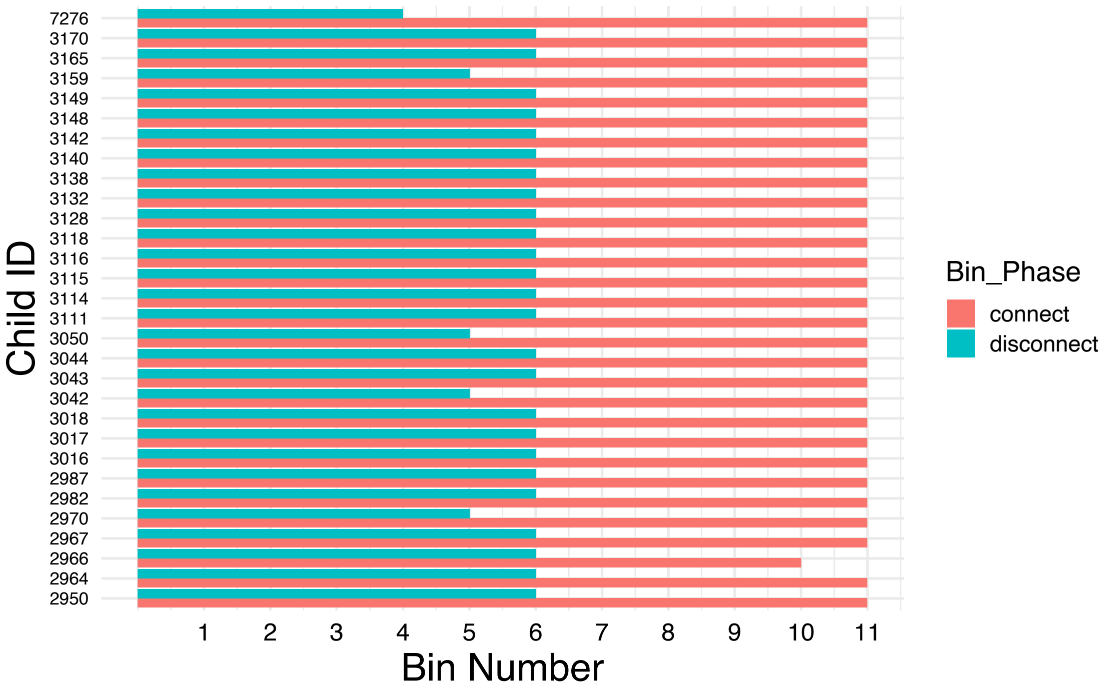

Figure S2. Individual Infants' Time Course of Vocalisation Count by Phase (Colour-Coded)

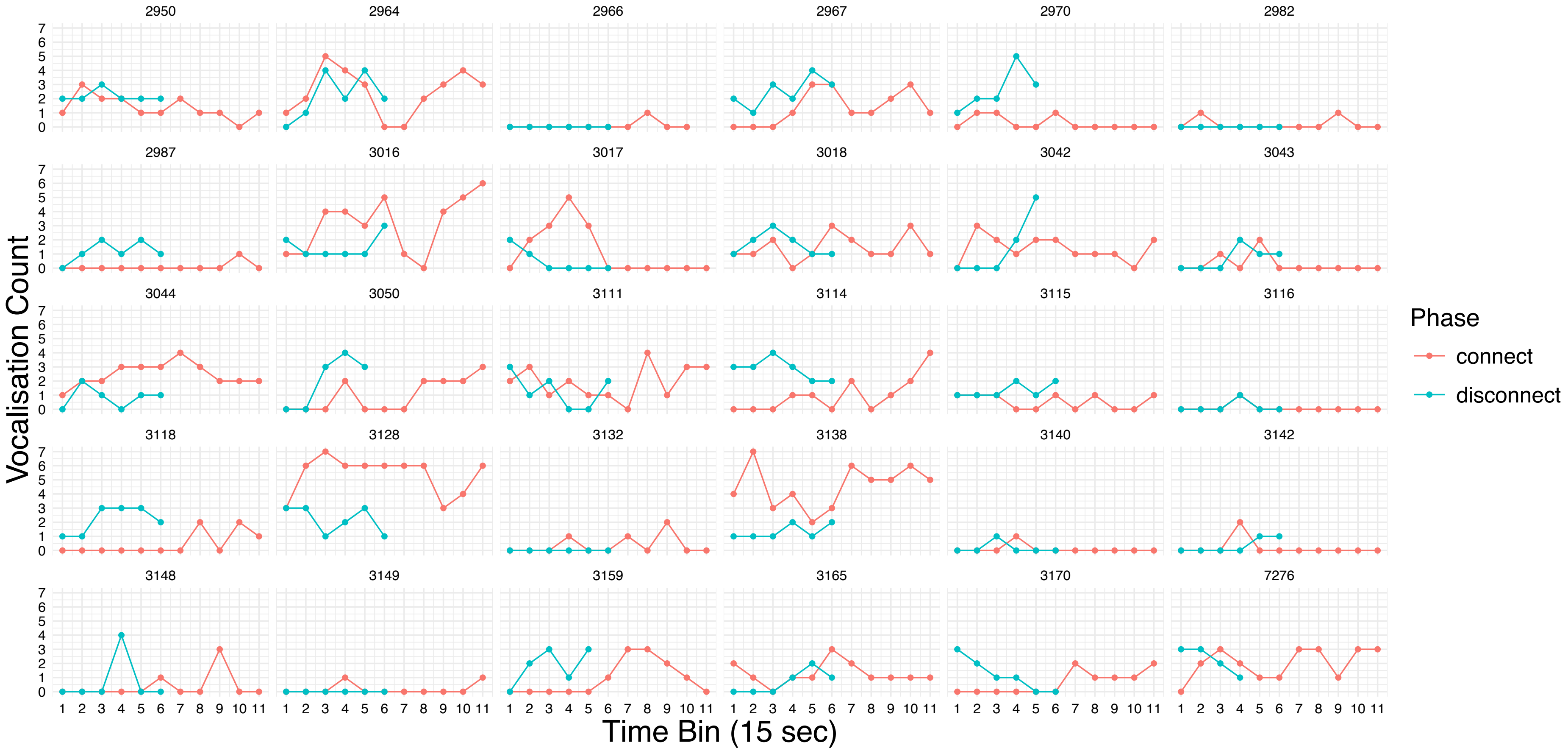

Table S1. Results of Generalised Linear Mixed Models Predicting Infants' Vocalisation Frequency in 15-Second Bins in the Disconnect Phase (Exploratory Model Including the Quadratic, But Not the Linear Term)

Predictors	*Incidence Rate Ratios*	*SE*	*CI*	*p*
(Intercept)	1.95	0.35	1.37 – 2.77	**<0.001**
Gender [Boy]	0.36	0.09	0.21 – 0.60	**<0.001**
Age days Z	0.99	0.13	0.77 – 1.29	0.968
Dur modelling Z	0.77	0.12	0.57 – 1.03	0.078
Prop contingency Z	1.18	0.18	0.87 – 1.59	0.291
Bin Number Quadratic	0.97	0.01	0.95 – 0.99	**0.017**
**Random Effects**				
σ^2	0.72			
τ_{00} Child_ID	0.26			
ICC	0.27			
N Child_ID	30			
Observations	174			
Marginal R^2 / Conditional R^2	0.278 / 0.472			

Comparing the Vocalisation Rate Per Minute Across Phases

The vocal extinction burst has been measured at different levels of granularity in prior research. At a coarse-grained level, it has been calculated as an overall increase in vocalisation rate per minute when contingent responsiveness is interrupted (Elmlinger et al., 2023; Goldstein et al., 2009; Venditti et al., 2024). We conduct this additional analysis for comparability. The mean vocalisation rate per minute was 4.90 (*SD* = 5.10) in the connect phase and 4.82 (*SD* = 3.17) in the disconnect phase. We fit a Linear Mixed Model to predict the vocalisation rate per minute as a function of phase, while controlling for gender, age, length of the adaptive modelling, and proportion of contingency. The likelihood ratio test was used to compare a null model including only control predictors to a full model, which also included the critical test predictor of phase. The fixed effects in the full model were i) phase

(connect vs. disconnect), which was dummy-coded with connect as the reference level, gender (dummy coded with girls as reference), and the z-transformed covariates of age (expressed in days), length of modelling (expressed in seconds), and proportion of contingency. The random effect structure included the random intercept of the subject. There was only one observation per condition (i.e., one vocalisation rate per phase per child). Therefore, the random slope was not theoretically identifiable and not included.

There was no meaningful effect of phase (connect vs. disconnect) on infants' vocalisation rate per minute. The likelihood ratio test indicated that the full model was not a significantly better fit to the data compared with the null model (χ^2 = 1.331, *df* = 1, *p* = .248). Model coefficients revealed that boys were associated with a reduced vocalisation rate compared to girls. We followed up on the identified gender difference with an exploratory interaction model, allowing for the effect of phase to vary across the levels of gender. Such an exploratory model was not a better fit for the data (χ^2 = 1.224, *df* = 2, *p* = .542). The results of the null, full, and exploratory models are reported in Table S2.

Table S2. Results of Linear Mixed Models Predicting Infants' Vocalisation Rate per Minute as a Function of Phase and Control Predictors

Predictors	*Estimates*	*SE*	*CI*	*p*	*Estimates*	*SE*	*CI*	*p*	*Estimates*	*SE*	*CI*	*p*
(Intercept)	0.30	0.20	-0.10 – 0.70	0.140	0.31	0.22	-0.14 – 0.76	0.174	0.14	0.25	-0.36 – 0.63	0.582
Gender [Boy]	-0.56	0.28	-1.12 – -0.00	**0.050**	-0.56	0.28	-1.12 – -0.00	**0.050**	-0.24	0.34	-0.93 – 0.45	0.493
Age days Z	-0.03	0.15	-0.32 – 0.26	0.850	-0.03	0.15	-0.32 – 0.26	0.850	-0.03	0.15	-0.32 – 0.26	0.850
Dur modelling Z	-0.29	0.16	-0.61 – 0.03	0.074	-0.29	0.16	-0.61 – 0.03	0.074	-0.29	0.16	-0.61 – 0.03	0.074
Prop contingency Z	0.20	0.15	-0.10 – 0.51	0.178	0.20	0.15	-0.10 – 0.51	0.178	0.20	0.15	-0.10 – 0.51	0.179
Phase [Disconnect]					-0.02	0.21	-0.43 – 0.39	0.918	0.32	0.29	-0.27 – 0.91	0.276
Phase [Disconnect] × Gender [Boy]									-0.65	0.40	-1.45 – 0.16	0.114
**Random Effects**												
σ^2	0.61				0.63				0.60			
τ_{00}	0.22 $_{Child\_ID}$				0.21 $_{Child\_ID}$				0.23 $_{Child\_ID}$			
ICC	0.26				0.25				0.27			
N	30 $_{Child\_ID}$				30 $_{Child\_ID}$				30 $_{Child\_ID}$			
Observations	60				60				60			

Marginal R^2 / Conditional R^2	0.225 / 0.430	0.223 / 0.416	0.245 / 0.451

Note. σ^2 = The variance of the residuals (errors) after accounting for both fixed and random effects; τ_{00} = The variance of the random intercepts, indicating the variability between subjects in their baseline levels; ρ_{01} = The correlation between random effects components; ICC = The proportion of total variance attributable to the grouping structure in the data.

Table S3. Results of Generalised Linear Mixed Models Predicting Infants' Vocalisation Frequency in 15-Second Bins (Including All Bins Irrespective of Bin Sample Size)

Predictors	*Incidence Rate Ratios*	*SE*	*CI*	*p*	*Incidence Rate Ratios*	*SE*	*CI*	*p*
(Intercept)	1.47	0.29	1.00 – 2.17	**0.049**	1.16	0.27	0.74 – 1.83	0.522
Gender [Boy]	0.44	0.11	0.27 – 0.72	**0.001**	0.43	0.10	0.27 – 0.69	**<0.001**
Age days Z	0.97	0.13	0.76 – 1.25	0.836	0.98	0.12	0.77 – 1.25	0.886
Dur modelling Z	0.78	0.11	0.59 – 1.03	0.080	0.80	0.11	0.61 – 1.05	0.103
Prop contingency Z	1.17	0.17	0.88 – 1.54	0.276	1.15	0.16	0.88 – 1.50	0.313
Bin Phase [disconnect]					1.57	0.30	1.09 – 2.27	**0.016**
Bin Number Linear					1.05	0.02	1.01 – 1.09	**0.008**
Bin Number Quadratic					0.99	0.01	0.98 – 1.00	0.296
Bin Phase [disconnect] × Bin Number Linear					0.96	0.03	0.89 – 1.02	0.175
Bin Phase [disconnect] × Bin Number Quadratic					0.98	0.01	0.96 – 1.01	0.193
**Random Effects**								
σ^2	0.47				0.47			
τ_{00}	0.85 $_{\text{Child\_ID}}$				0.91 $_{\text{Child\_ID}}$			
τ_{11}	0.56 $_{\text{Child\_ID.Bin\_Phasedisconnect}}$				0.53 $_{\text{Child\_ID.Bin\_Phasedisconnect}}$			
ρ_{01}	-0.81 $_{\text{Child\_ID}}$				-0.86 $_{\text{Child\_ID}}$			

ICC	0.64	0.57
N	30 $_{Child\_ID}$	30 $_{Child\_ID}$
Observations	572	572
Marginal R^2 / Conditional R^2	0.168 / 0.703	0.206 / 0.661

Note. σ^2 = The variance of the residuals (errors) after accounting for both fixed and random effects; τ_{00} = The variance of the random intercepts, indicating the variability between subjects in their baseline levels; ρ_{01} = The correlation between random effects components; ICC = The proportion of total variance attributable to the grouping structure in the data.

Table S4. Results of Generalised Linear Mixed Models Predicting Infants' Vocalisation Frequency in 15-Second Bins (Excluding Infants Experiencing <25% Contingency)

Predictors	*Incidence Rate Ratios*	*SE*	*CI*	*p*	*Incidence Rate Ratios*	*SE*	*CI*	*p*
(Intercept)	1.65	0.32	1.13 – 2.41	**0.010**	1.29	0.33	0.78 – 2.12	0.317
Gender [Boy]	0.55	0.16	0.31 – 0.99	**0.045**	0.55	0.16	0.32 – 0.96	**0.035**
Age days Z	0.88	0.12	0.68 – 1.15	0.344	0.89	0.12	0.69 – 1.14	0.360
Dur modelling Z	0.78	0.11	0.58 – 1.04	0.085	0.79	0.11	0.60 – 1.04	0.094
Prop contingency Z	0.78	0.18	0.49 – 1.22	0.271	0.79	0.18	0.51 – 1.22	0.288
Bin Phase [disconnect]					1.53	0.32	1.01 – 2.31	**0.046**
Bin Number Linear					1.05	0.02	1.01 – 1.09	**0.025**
Bin Number Quadratic					1.00	0.01	0.98 – 1.01	0.425
Bin Phase [disconnect] × Bin Number Linear					0.88	0.09	0.71 – 1.09	0.240
Bin Phase [disconnect] × Bin Number Quadratic					0.95	0.03	0.90 – 1.01	0.112
**Random Effects**								
σ^2	0.42				0.42			
τ_{00}	0.96 $_{\text{Child\_ID}}$				0.98 $_{\text{Child\_ID}}$			
τ_{11}	0.62 $_{\text{Child\_ID.Bin\_Phasedisconnect}}$				0.60 $_{\text{Child\_ID.Bin\_Phasedisconnect}}$			

ρ_{01}	-0.88 $_{Child\_ID}$	-0.90 $_{Child\_ID}$
ICC	0.70	0.63
N	24 $_{Child\_ID}$	24 $_{Child\_ID}$
Observations	401	401
Marginal R^2 / Conditional R^2	0.118 / 0.732	0.159 / 0.687

Note. σ^2 = The variance of the residuals (errors) after accounting for both fixed and random effects; τ_{00} = The variance of the random intercepts, indicating the variability between subjects in their baseline levels; ρ_{01} = The correlation between random effects components; ICC = The proportion of total variance attributable to the grouping structure in the data.

Table S5. Results of Generalised Linear Mixed Models Predicting Infants' Vocalisation Frequency in 15-Second Bins (Excluding the First Bin of the Connect Phase)

Predictors	*Incidence Rate Ratios*	*SE*	*CI*	*p*	*Incidence Rate Ratios*	*SE*	*CI*	*p*
(Intercept)	1.49	0.32	0.99 – 2.26	0.058	1.19	0.28	0.75 – 1.90	0.462
Gender [Boy]	0.39	0.11	0.22 – 0.68	**0.001**	0.38	0.10	0.22 – 0.65	**<0.001**
Age days Z	0.98	0.14	0.74 – 1.28	0.867	0.98	0.13	0.76 – 1.28	0.890
Dur modelling Z	0.74	0.12	0.54 – 1.01	0.054	0.75	0.11	0.56 – 1.01	0.061
Prop contingency Z	1.20	0.19	0.88 – 1.64	0.251	1.18	0.18	0.87 – 1.59	0.280
Bin Phase [disconnect]					1.53	0.30	1.04 – 2.25	**0.030**
Bin Number Linear					1.00	0.03	0.95 – 1.05	0.916
Bin Number Quadratic					1.01	0.01	0.99 – 1.02	0.294
Bin Phase [disconnect] × Bin Number Linear					0.93	0.10	0.76 – 1.14	0.475
Bin Phase [disconnect] × Bin Number Quadratic					0.95	0.03	0.89 – 1.00	0.058
**Random Effects**								
σ^2	0.51				0.51			
τ_{00}	0.84 $_{Child\_ID}$				0.90 $_{Child\_ID}$			
τ_{11}	0.54 $_{Child\_ID.Bin\_Phasedisconnect}$				0.53 $_{Child\_ID.Bin\_Phasedisconnect}$			
ρ_{01}	-0.77 $_{Child\_ID}$				-0.82 $_{Child\_ID}$			

ICC	0.62	0.57
N	30 $_{Child\_ID}$	30 $_{Child\_ID}$
Observations	473	473
Marginal R^2 / Conditional R^2	0.211 / 0.703	0.236 / 0.673

Note. σ^2 = The variance of the residuals (errors) after accounting for both fixed and random effects; τ_{00} = The variance of the random intercepts, indicating the variability between subjects in their baseline levels; ρ_{01} = The correlation between random effects components; ICC = The proportion of total variance attributable to the grouping structure in the data.
